# Identification and Characterization of Mutations in Ubiquitin Required for Non-covalent Dimer Formation

**DOI:** 10.1016/j.str.2019.06.008

**Published:** 2019-09-03

**Authors:** Mads Gabrielsen, Lori Buetow, Dominika Kowalczyk, Wei Zhang, Sachdev S. Sidhu, Danny T. Huang

**Affiliations:** 1 Cancer Research UK Beatson Institute, Institute of Cancer Sciences, University of Glasgow, Garscube Estate, Switchback Road, Glasgow G61 1BD, UK; 2 Donnelly Centre for Cellular and Biomolecular Research, Banting and Best Department of Medical Research, University of Toronto, 160 College Street, Toronto, ON M5S 3E1, Canada

**Keywords:** ubiquitin, monomer, dimer, non-covalent, ubiquitin variants, X-ray crystallography, SAXS

## Abstract

Ubiquitin (Ub) is a small protein that post-translationally modifies a variety of substrates in eukaryotic cells to modulate substrate function. The ability of Ub to interact with numerous protein domains makes Ub an attractive scaffold for engineering ubiquitin variants (UbVs) with high target specificity. Previously, we identified a UbV that formed a non-covalent stable dimer via a β-strand exchange, and in the current work we identified and characterized the minimal substitutions in the primary sequence of Ub required to form a higher ordered complex. Using solution angle scattering and X-ray crystallography, we show that a single substitution of residue Gly10 to either Ala or Val is sufficient to convert Ub from a monomer to a dimer. We also investigate contributions to dimer formation by the residues in the surrounding sequence. These results can be used to develop next-generation phage-display libraries of UbVs to engineer new interfaces for protein recognition.

## Highlights

• G10V substitution converts ubiquitin from a monomer to a dimer
• Ubiquitin monomer and dimer adopt different conformations in solution
• Crystal structure of ubiquitin dimer reveals the molecular basis of dimerization
• Dimeric ubiquitin has rotational flexibility along the dimerization arm

## Graphical Abstract

**Figure:**
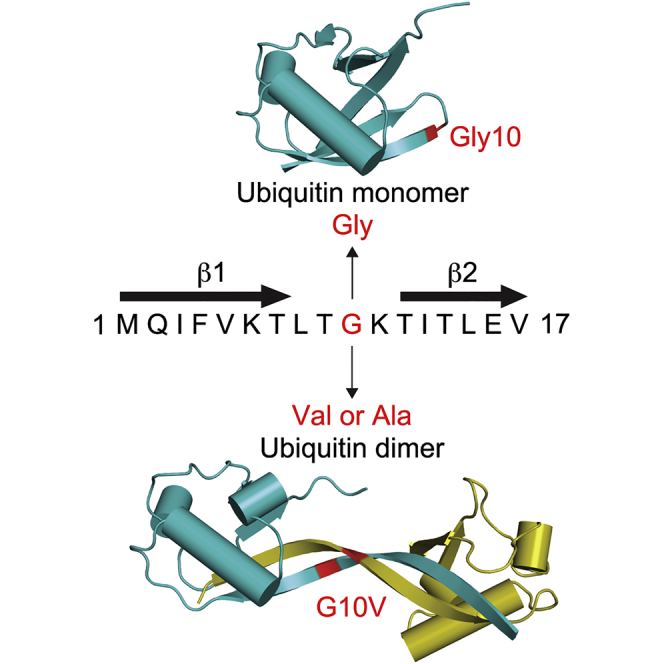

## Teaser

Ubiquitin scaffold libraries have been used to identify affinity reagents for a broad range of targets. To expand library diversity, Gabrielsen et al. investigate the minimal requirements for converting ubiquitin from a monomer to a non-covalent dimer based on a dimeric ubiquitin variant with multiple substitutions identified previously.

## Introduction

Ubiquitin (Ub) is a small 76-amino-acid protein that functions as a post-translational modifier on a wide range of substrate proteins in the eukaryotic cell (Hershko and Ciechanover, 1998). The process of ubiquitination is initiated by E1, which uses Mg^2+^-ATP to activate Ub and conjugate it to an E2 Ub-conjugating enzyme via a thioester bond between the C-terminal diglycine on Ub and a catalytic cysteine on the E2. Ub from this E2∼Ub complex (∼ denotes the thioester bond) is then transferred to a substrate in a reaction mediated by an E3 ligase, thereby forming an isopeptide bond between the C-terminal glycine of Ub and a free amine group from a substrate lysine or N-terminal methionine (Komander and Rape, 2012, Pickart and Eddins, 2004). E3 ligases are divided into three families depending on the Ub transfer mechanism (Buetow and Huang, 2016)—HECT, RING, and RING between RING—and of these three, RING E3s are the most abundant (Deshaies and Joazeiro, 2009). Ub also associates with many proteins via non-covalent interactions, and a wide range of proteins have Ub-associated domains and Ub-binding domains to facilitate this type of interaction (Dikic et al., 2009).

**Figure 1:**
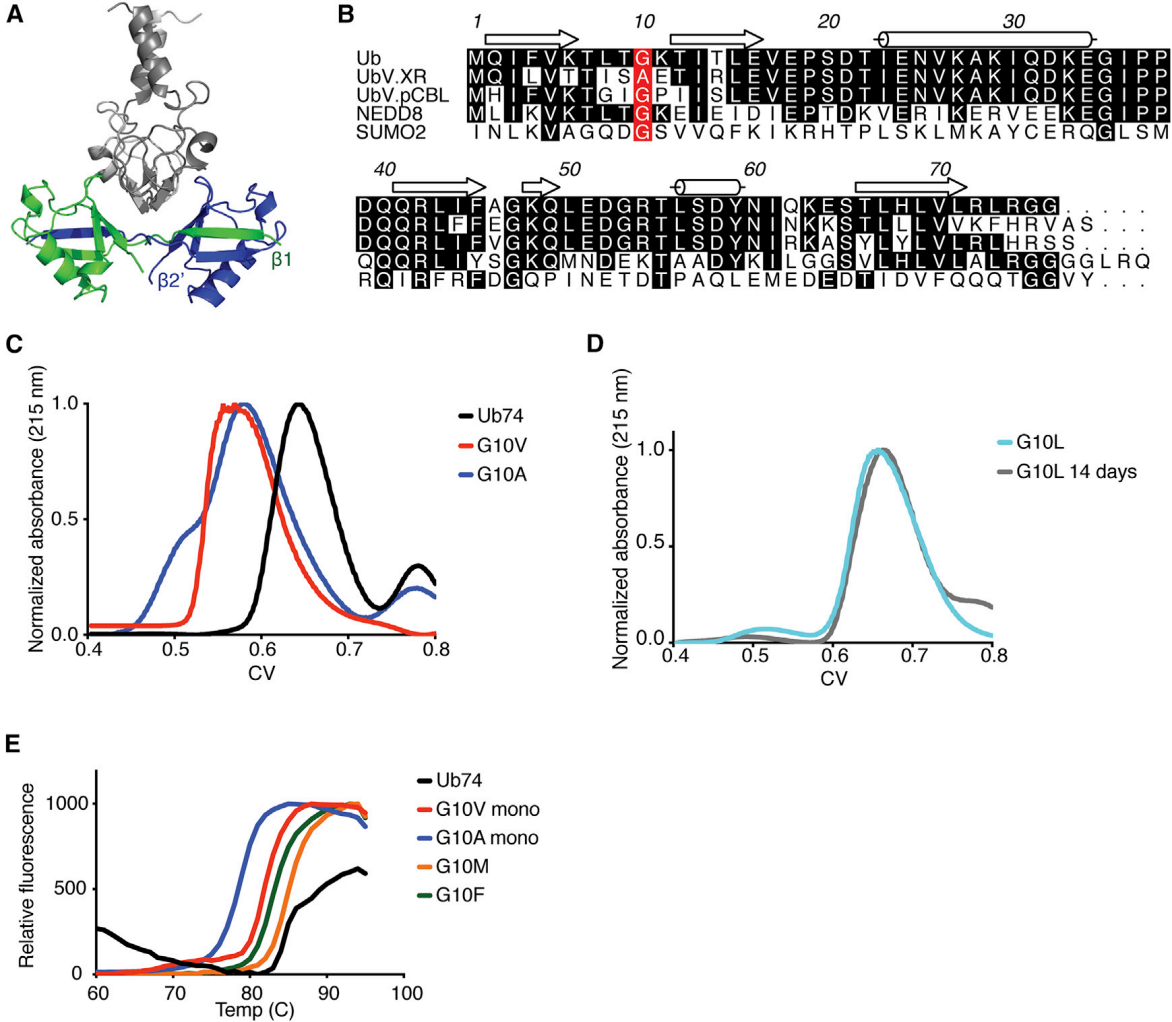
Substitution of Gly10 to Ala or Val in Ub74 Promotes Dimerization (A) Crystal structure of XIAP RING (gray) and dimeric UbV.XR (green and blue) (PDB: 5O6T). (B) Sequence alignments of ubiquitin (Ub), UbV.XR, and UbV.pCBL (Gabrielsen et al., 2017) with NEDD8-Ubl and SUMO2-Ubl created with ALINE (Bond and Schüttelkopf, 2009). Numbering is based on ubiquitin, and identical residues are highlighted in black. Residue 10 is highlighted in red. (C) Normalized analytical size-exclusion chromatograms of Ub74 and variants with substitutions of Gly to Ala or Val in position 10. The profiles of Ub74 G10A and G10V have peaks that elute at volumes consistent with a dimer. See also Figure S1A. (D) Normalized analytical size-exclusion chromatograms of Ub74 G10L immediately following purification and after incubation at 4°C for 14 days. There is no shift from monomer to dimer. See also Figures S1B and S1C. (E) Stability of Ub74 Gly10 variants as measured in a thermofluorescence assay. The monomeric fractions of Ub74 G10A and G10V have lower melting points than Ub74, Ub74 G10F, and Ub74 G10M. Assays were performed in triplicate, and melting points and standard error were calculated using a Boltzmann sigmoid fit in GraphPad Prism. The curves shown are from one set of assays.

Because Ub uses a common surface to interact with a wide variety of proteins with relatively low affinity, it is an attractive scaffold for engineering variants with greater specificity for select targets (Ernst et al., 2013, Leung et al., 2017). Previously, we generated a ubiquitin variant (UbV) library (Ernst et al., 2013) and used phage display to select UbVs that target and modulate the activities of multiple classes of proteins including deubiquitinases (DUBs), HECT E3 and RING E3 ligases, and Ub-interacting motifs (Brown et al., 2016, Gabrielsen et al., 2017, Gorelik et al., 2016, Manczyk et al., 2017, Zhang et al., 2016, Zhang et al., 2017a, Zhang et al., 2017b). Using this method, we unexpectedly identified a dimeric UbV that is selective for the RING domain of XIAP (hereafter referred to as XR) (Gabrielsen et al., 2017). In crystal structures of this UbV alone and bound to XR, β1 and β1′ from the two UbV chains are swapped, forming an elongated strand that fulfills all hydrogen-bonding criteria required to complete the β-sheet criteria (Figure 1A) that occur between β1 and β2 in wild-type Ub. When purified by size-exclusion chromatography, this XR-selective UbV, herein termed UbV.XR, was present in two populations: one corresponding to the molecular weight of a dimer and the other corresponding to that of a monomer. Over time, there was a shift in the population from monomeric to dimeric UbV.XR, and the dimer population was eliminated by reverting Ala10 in UbV.XR to Gly, the corresponding residue in wild-type Ub.

In addition to position 10, Ub and UbV.XR differ at a number of other positions in β1 and the loop between β1 and β2, which could influence dimerization. In this work, we have determined the minimum substitutions required to convert wild-type Ub from a stable monomer to a dimer by introducing point mutations in β1 and the loop between β1 and β2. This not only provides a fundamental understanding of Ub protein characteristics, but will allow us to design dimeric libraries of UbVs to expand our target space for developing selective modulators of cellular activity for Ub-binding proteins.

## Results

### Substitution of Gly10 Is Sufficient to Promote Oligomerization

Of the many structures of UbVs in complex with their binding partners determined to date, only UbV.XR has been reported to exist as a dimer. Sequence and structural comparisons of Ub with the UbVs that have been crystallized suggest that substitution of Gly10 might be sufficient to promote Ub dimerization (Figure 1B). In UbV.XR, Ala10 was shown to be crucial for dimerization. When this residue was reverted to Gly, the variant remained monomeric even when left at 4°C for a week whereas the UbV.XR Ala10 monomer population shifted toward dimer in the same time frame (Gabrielsen et al., 2017). However, in UbV.XR, there are a number of additional amino acid substitutions in this region that have not been excluded from having a role in UbV.XR dimerization. To determine whether a single substitution of residue Gly10 in Ub promotes oligomerization, we introduced Gly10 substitutions of increasing size into a construct of Ub lacking the C-terminal diglycine motif (Ub74). Using gel-filtration chromatography, we assessed whether substituting Gly10 in Ub74 with Ala, Val, Leu, Met, Phe, or Arg promoted dimer formation (Figures 1C and S1A) and found that the Ala and Val substitutions existed in two populations that eluted at volumes consistent with the molecular weights of a Ub monomer and dimer. When left at 4°C for a week, a fraction of the monomer at ∼10 mg/mL slowly converted to a mix of monomeric and dimeric Ub, whereas when left for a week, a fraction of the dimer at ∼10 mg/mL did not revert to monomer (Figures S1B and S1C). When Gly10 was substituted with residues with larger side chains, little evidence of dimer formation was observed, even after a 2-week incubation at 4°C as shown for Ub74 G10L (Figure 1D). We also measured melting temperatures of Ub74 and the position-10 variants using dynamic fluorescence scanning to assess stability. While Ub74 and Ub74 G10M had melting temperatures of 85.5°C ± 0.2°C and 86.3°C ± 0.2°C, respectively, the monomeric fractions of variants with Ala and Val substitutions had melting temperatures of 79.9°C ± 0.7°C and 81.9°C ± 0.2°C, respectively, showing that these two variants are less stable (Figure 1E). However, the Ub74 G10F substituted sample had a melting temperature of 84.3°C ± 0.3°C yet remained monomeric. These data show that factors other than mutation-induced instability may contribute to non-covalent dimer formation by Ub.

### Ub74 and Ub74 G10V Have Distinct Shapes in Solution

**Figure 2:**
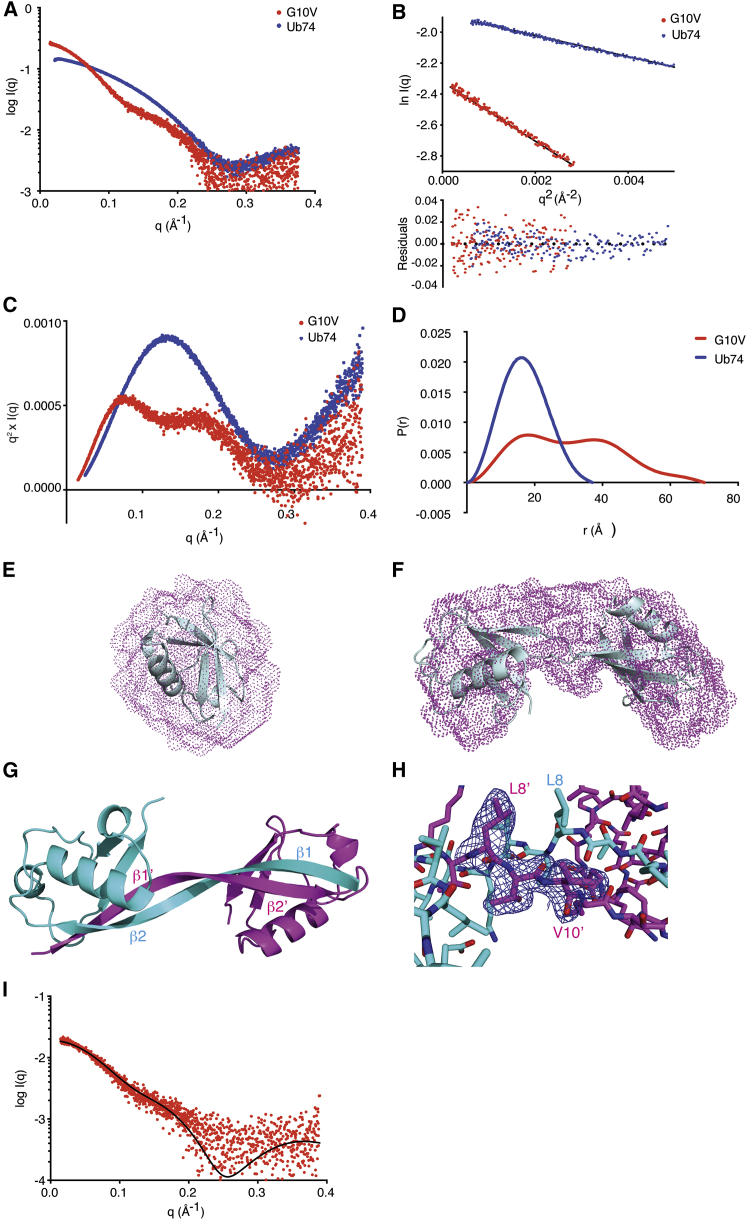
Ub74 G10V and Ub74 Adopt Different Conformations (A) Scaled experimental intensity plots of Ub74 (blue) and Ub74 G10V (red) show a clear difference in the conformations of the two samples in solution. The colors representing each sample are consistent throughout the figure. (B) Guinier plot of Ub74 and Ub74 G10V with residuals shown below. *R*_g_ values of 14.4 Å and 23.9 Å were derived from these data for Ub74 and Ub74 G10V, respectively. (C) Kratky plots of Ub74 and Ub74 G10V, highlighting the fundamental differences between the two proteins in solution. (D) Relative pair distribution function (P(r)) of Ub74 with a *D*_max_ of 44 Å and Ub74 G10V with a *D*_max_ of 76 Å. (E) *Ab initio* reconstruction of Ub74 superposed on the crystal structure of Ub from PDB: 1UBQ. (F) *Ab initio* reconstruction of Ub74 G10V superposed on the crystal structure from this study. (G) Crystal structure of Ub74 G10V dimer, showing how β1 has exchanged position with β1′ from the partnering subunit. Note that the overall Ub fold is maintained for both chains. (H) Close-up of G10V and the β1β2 loop with Polder map contoured at 2σ. (I) Solution scattering of Ub74 G10V, fitted with the theoretical scattering curve of the crystal structure of Ub74 G10V.

To confirm observations from our gel-filtration analyses, we analyzed Ub74 and the dimer fraction of Ub74 G10V using small-angle X-ray scattering (SAXS). The intensity and Kratky plots are dramatically different for the two samples (Figures 2A and 2B), suggesting that a single substitution of Gly10 to Val drastically alters the size and shape of Ub. The radius of gyration (*R*_g_) and maximum distance (*D*_max_) were 14.4 Å and 44 Å for Ub74 and 23.9 Å and 76 Å for Ub74 G10V (Figures 2C and 2D), respectively; these clearly show that Ub74 G10V forms a larger complex in solution than Ub74. The *D*_max_ and *R*_g_ of Ub74 G10V appear to be concentration independent, suggesting that the dimeric state does not change with protein concentration within our tested range (6.25–50 mg/mL). Additionally, when we calculated *ab initio* models of Ub74 G10V and Ub74 based on the experimental data collected, the calculated bead models were distinctly different (Figures 2E and 2F), and when high-resolution structures of Ub are superposed on the envelopes, the Ub74 G10V envelope accommodates two molecules of Ub and the Ub74 envelope one. Together these data show that Ub74 and Ub74 G10V adopt different conformations in solution consistent in size with a monomer and dimer, respectively.

### Molecular Structure of Ub74 G10V

To investigate whether Ub74 G10V resembled the UbV.XR dimer, we determined the structure to a resolution of 2.23 Å from crystals grown from the dimeric gel-filtration fractions. There are 12 chains in the asymmetric unit forming six dimers, in which the β1 strands from both subunits of the dimer are swapped and complete the β sheet in the partnering subunit (Figure 2G and Table 1) as observed in UbV.XR.

**Table 1:** Data Collection and Refinement Statistics

	Ub74 G10V
**Data Collection**	

PDB ID	6QK9
Space group	*P*2_1_2_1_2_1_
Cell dimensions	
*a*, *b*, *c* (Å)	83.93, 87.26, 109.84
α, β, γ (°)	90, 90, 90
Resolution (Å)	29.16–2.23 (2.29–2.23)^a^
*R*_merge_ (%)	9.3 (76.4)
*R*_pim_ (%)	5.8 (48.5)
Completeness (%)	99.4 (93.1)
Multiplicity	6.6 (6.1)
*I*/σ*I*	13.2 (2.0)
CC_1/2_	0.98 (0.85)
Wilson *B* (Å^2^)	34.7

**Refinement**	

*R*_work_ (%)	23.90
*R*_free_ (%)	28.1
No. of atoms	
Protein	13,886
Water	204
RMSD bond	0.010
RMSD angle	1.321
*B* factors	
Main chain	40.4
Side chain	49.9
Ramachandran favorable (%)	99.65
Additional allowed (%)	0.35

a Values in parentheses are for highest-resolution shell.

Polder maps (Liebschner et al., 2017) (Figure 2H) confirm that the electron density for the β1β2 loop clearly extends between two adjacent chains such that β1 interacts with β2′ on a neighboring chain to form a β sheet. Additionally, there are no significant differences between the *B* factors of the atoms involved in the β1 exchange and the remainder of the atoms in each molecule of the dimer. The quality of chains D and H (part of dimers CD and GH, respectively) is poorer than that of the others, reflected by the higher average *B* factors of these chains (72 and 62 Å, respectively) when compared with the overall average *B* factor of all chains (51 Å). The complementary chains in each dimer pair have a complete elongated β1 strand.

Comparison of the crystal structure and solution studies of Ub74 G10V suggests that the dimer from the crystal structure is also present in solution. A theoretical scattering curve calculated from the crystal structure fits well to the solution data with a χ^2^ value of 3.8 compared with the crystal structure of Ub74, which fits with a χ^2^ of 20.8 (Figure 2I), and the crystal structure of the Ub74 G10V dimer was easily superposed with the envelope calculated from the solution scattering data (Figure 2F). In addition, the calculated *R*_g_ of the Ub74 G10V crystal structure is 21.5 Å (CRYSOL), fitting closely with the experimentally obtained *R*_g_. In comparison, the calculated *R*_g_ of monomeric Ub from a crystal structure of Ub (1UBQ) and experimentally determined *R*_g_ from Ub74 are 13.6 and 14.0 Å, respectively. These data suggest that the Ub74 G10V dimer is not merely a crystallographic artifact.

The dimer interface comprises around 1,800 Å^2^ of an overall accessible surface area of ∼6,000 Å^2^ for each subunit. There appears to be a certain degree of freedom around the dimerization arm, producing two distinct populations of dimers in the asymmetric unit. The first population consists of the chain pairs AB and IJ, which superpose with a root-mean-square deviation (RMSD) of 1.55 Å for 2,139 atoms, and the second population consists of chain pairs EF, KL, and GH, which all superpose with an RMSD of less than 0.8 Å for ∼2,000 atoms. When chain pairs from the two populations are superposed, the RMSD increases to ∼2.4 Å. Chain pair CD sits in a conformation between the two populations, superposing to AB and EF with RMSDs of 2.3 and 1.48 Å, respectively, highlighting the flexibility of the dimerization arm.

**Figure 3:**
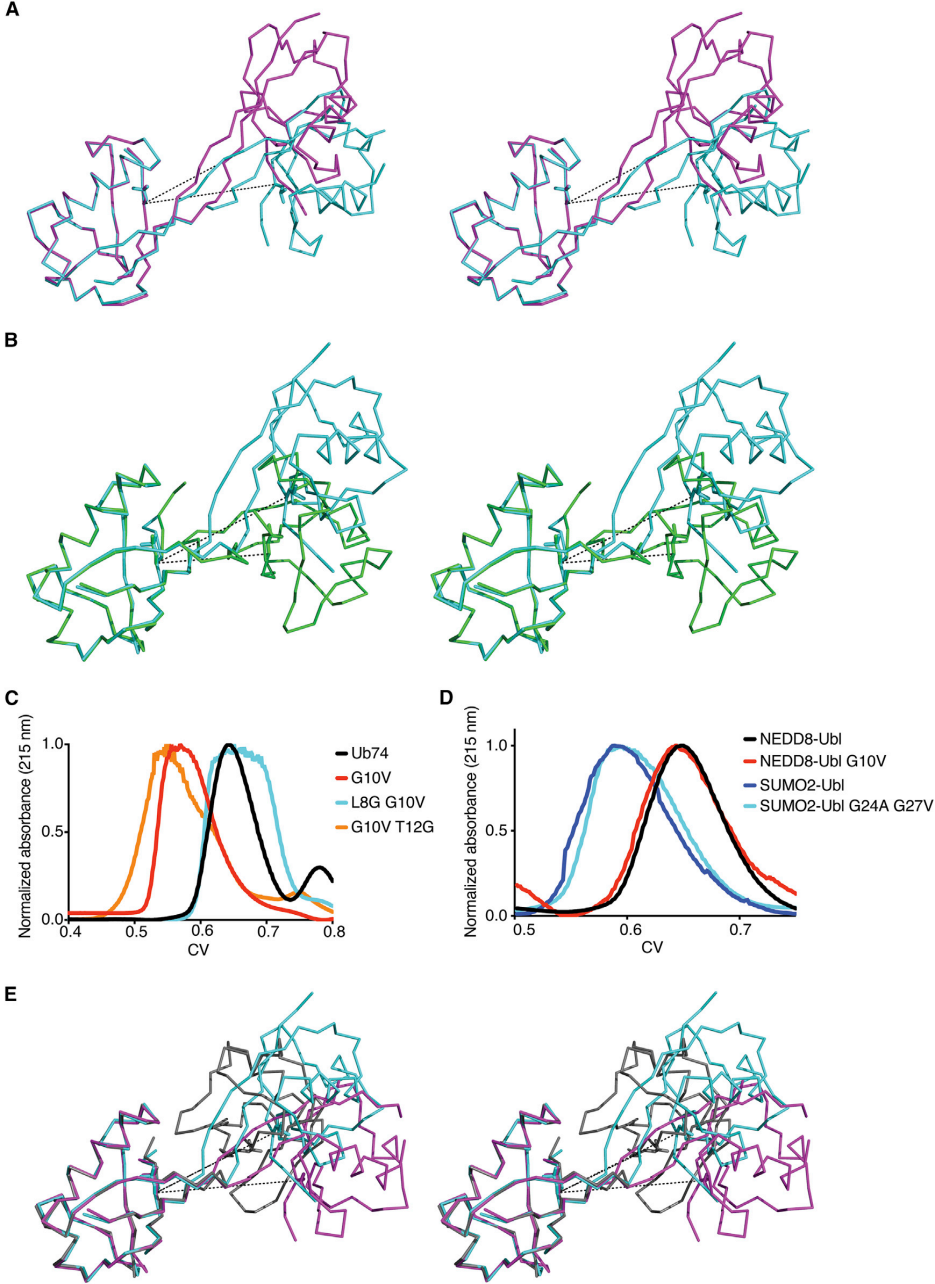
Dimerization Criteria (A) Superposed Cα-ribbon traces of chain AB (magenta) and chain EF (cyan) dimers from the two populations in the asymmetric unit of the crystal structure of Ub74 G10V, in wall-eye stereo. Leu69 from each subunit is shown as sticks and the dotted line shows the distance measured. See also Figure S1D. (B) Superposed Cα-ribbon traces of UbV.XR alone (green, PDB: 5O6S) and Ub74 G10V (cyan). Leu69 from each subunit is shown as sticks. (C) Normalized analytical size-exclusion chromatogram of Ub74 G10V and Ub74 compared with variants in which other residues in the β1β2 loop have been individually replaced with Gly. See also Figures S1E and S1F. (D) Normalized analytical size-exclusion chromatogram of NEDD8-Ubl, NEDD8-Ubl G10V, SUMO2-Ubl, and SUMO2-Ubl G24A G27V. There is no evidence of dimer formation in any of these Ubl variants. (E) As in (A) but including the K48-linked diUb from PDB: 5MN9, colored gray.

The main source of divergence between these two populations arises due to differences in rotation between the two Ub bodies in the dimer subunits. To compare the compactness of the dimer populations, we measured the distance between Leu69 in both subunits, which sits near the dimer interface, and found that both populations of dimer subunits are separated by a distance of about 20 Å for AB, 21 Å for CD, and 22 Å for EF (Figure 3A). Furthermore, we measured the angle formed between Asp39, Val10, and Asp39′ to describe the rotation between the two molecules (Figure S1D). The populations differ quite dramatically, with angles of 144° for AB, 159° for CD, and 163° for EF. While these differences are obvious in the crystal lattice, theoretical scattering profiles calculated for each dimer configuration show no detectable differences between the two forms in solution. When compared with the published UbV.XR dimer structure, differences in rotation between the two Ub bodies are evident (Figure 3B), highlighting the flexibility in UbV dimer.

### The β1β2 Loop Is Important for the Stability of Ub

Because substitution of G10 to Val changes the oligomeric state of Ub from a monomer to a dimer, we set out to determine whether the monomer could be recovered if a glycine was introduced elsewhere in the β1β2 loop (Figure 1B). Initially, residues Leu8 and Thr12 were substituted with glycine in Ub74 G10V, and analyzed by analytical size-exclusion chromatography as described above. Ub74 L8G G10V was monomeric, whereas Ub74 G10V T12G was dimeric (Figure 3C). To expand on the borders of where the glycine substitution can occur and still recover the monomeric state, we made a range of individual substitutions with glycine in Ub74 G10V, namely Phe4, Val5, Lys6, Thr7, Lys11, Ile13, Thr14, or Leu15. Ub74 G10V with V5G or T7G substitutions did not express soluble protein. When Phe4 or Lys6 was substituted with glycine in Ub74 G10V, the protein remained a dimer (Figure S1E). While a Lys11 substitution with Gly was monomeric, when Ile13, Thr14, or Leu15 was substituted with Gly in Ub74 G10V, the protein was predominantly a dimer (Figure S1F). Based on these gel-filtration profiles, our data suggest that a Gly substitution within the β1β2 loop of Ub74 G10V is sufficient to maintain the monomeric state of Ub; however, a Gly substitution in neighboring residues in β1 or β2 does not affect dimer formation caused by substitutions in Gly10.

### Dimerization via a Single Mutation Is Unique to Ub

Structure-based sequence alignments with a number of ubiquitin-like proteins (Ubls) revealed that several contained Gly residues in positions corresponding to Gly10 in Ub. We introduced Val substitutions at these positions in two Ubls derived from NEDD8 and SUMO2, and investigated their oligomeric state by size-exclusion chromatography (Figure 3D). No dimer formation was apparent in either Val-substituted Ubl although there was a marked reduction in solubility of the NEDD8-Ubl variant. Because the SUMO2-Ubl contains a second Gly in the loop between β1 and β2 that might potentially compensate for any inflexibility introduced as observed in Ub, we also analyzed the SUMO2-Ubl G24A G27V double mutant using gel-filtration chromatography and found no evidence of oligomerization (Figure 3D). These data suggest that other surrounding residues contribute to the stability of Ubl folds, and that further mapping of these regions is required to ascertain how these residues contribute to dimer formation and stability.

## Discussion

Ub scaffold libraries have been used to develop affinity reagents for a broad range of applications including probing cell signaling networks, elucidating protein function, and delivering targeted biotherapeutics (Brown et al., 2016, Gabrielsen et al., 2017, Gorelik et al., 2016, Job et al., 2015, Lorey et al., 2014, Manczyk et al., 2017, Zhang et al., 2016, Zhang et al., 2017a, Zhang et al., 2017b). Generating new search spaces relies on increasing the diversity of the Ub scaffold libraries. In a previous study, we serendipitously identified UbV.XR, which self-associates to form a stable non-covalent dimer and only binds its target as a dimer. In UbV.XR, Ala10 in the β1β2 loop is essential for dimerization but whether there are contributions from other mutations in this region is unknown. To facilitate the use of this non-covalent dimer conformation as a scaffold to generate additional UbV libraries, we investigated substitution requirements in the β1β2 loop of Ub to convert it to a dimer. We found that a single substitution of Gly10 with Ala or Val is sufficient to induce dimerization of Ub and that Ub74 G10V reverts to a monomeric state when certain other residues from the β1β2 loop are substituted with Gly. A crystal structure of Ub74 G10V shows that the dimer interface resembles that of UbV.XR, and SAXS analyses suggest this dimer conformation is also present in solution. Substitution of Gly to Val in the β1β2 loop of NEDD8-Ubl or SUMO2-Ubl does not promote dimerization, demonstrating that this mechanism for dimer formation is specific to Ub.

Of the Gly10 substitutions tested (Ala, Val, Leu, Met, Phe, or Arg), only Val and Ala promoted dimer formation. Interestingly, the monomeric populations of each of these variants had lower melting point temperatures than either wild-type Ub or the other UbVs. This mutation-induced instability might be a factor that allows β1 to detach from the β sheet in the globular body of the monomer to form the dimer. Once formed, the dimer appears to be very stable, as there was no evidence of dissociation to a monomer. However, Ub74 G10V reverted to monomer when other residues in the β1β2 loop were substituted with glycine, suggesting that the presence of a Gly in this loop plays a critical role in Ub's ability to form a non-covalent domain-swapped dimer. Nevertheless, our data show that this non-covalent dimerization is only observed in Ub and not other Ubls despite their high structural conservation; hence, it is likely that other factors also play a role in dimerization.

What drives dimer formation is as yet unclear. Despite ubiquitin being a compact globular protein, it has several flexible regions including the C-terminal tail that is essential for conjugation and the β1β2 loop (Gladkova et al., 2017). We hypothesize that two steps occur in the formation of Ub74 G10A or G10V dimers: these Gly10 mutations promote instability, allowing β1 to detach from β2, and, while the strand is dislodged, it interacts with a similarly β1-detached neighboring molecule to form a dimer. Another dimeric UbV selective for the deubiquitinase USP15 exhibits the same strand exchange as observed in Ub74 G10V (Teyra et al., 2019); although there is no substitution of Gly10, a loop insertion is present between residues Leu8 and Thr9 that may account for the required destabilization. Whether there are other factors that contribute to dimer formation or whether there is a threshold for stability is unclear. The melting temperature of Ub74 G10F is higher than that of Ub74 G10V or G10A but lower than that of Ub74, and in cases such as this, two free β1-strand molecules may not encounter one another so it is energetically more favorable to return to a native state.

There are a number of diUb structures available in the PDB that are formed from linkages involving Lys side chains or linear Met1 chains. The overall shape of the Ub74 G10V dimer and the various diUbs appear similar when superposed, as they comprise two globular proteins connected by a short linker consisting of the C-terminal tail and a lysine side chain, or by the elongated β1 observed in Ub74 G10V. However, superposition of one Ub chain from each structure reveals that the two Ub chains relate to each other through a wide range of angles. The closest structural arrangement to Ub74 G10V is the open conformation of K48-linked diUb from 5MN9 in which the diUb is part of a complex with a fragment of the DUB MINDY-1/FAM63A. In both Ub74 G10V and this K48-linked diUb, the distance between Leu69 and Leu69′ is 22 Å (Figure 3E).

Based on rotational conformational differences between the two Ub bodies of dimeric subunits within the asymmetric unit, our Ub74 G10V dimer structure suggests that the dimerization arm is flexible. This feature was not observed in our prior UbV.XR structure, but when UbV.XR is bound to XR it becomes more compact and the distance between the Leu69 in each UbV.XR decreases from 20 to 15 Å (Gabrielsen et al., 2017). Interactions with XR appear to stabilize the dimerization arm in UbV.XR and increase the dimer compactness, suggesting that the Ub dimer may also become more compact upon binding to another protein.

The discovery of dimeric UbV.XR and its stimulatory effect upon its target, XIAP RING, suggests there is scope to expand upon the diversity covered by current phage-displayed UbV libraries. In this study, we set out to elucidate which Ub residues require substitution to form a dimeric scaffold and found that substitution of Gly10 to Val or Ala was sufficient, provided no other Gly substitutions are introduced in the β1β2 loop. These substitutions can be used to develop next-generation libraries that will encode dimeric UbVs with novel interfaces for protein recognition. There is increased interest in developing dimeric UbVs, as these may allow improved specificity and binding and exploit bivalent avidity effects. Recently, Teyra et al. (2019) developed linear dimeric UbVs selective for the deubiquitinase USP15 by linking together UbVs that bind tandem domains of this enzyme. These dimeric UbVs show an increased binding to and inhibition of the deubiquitinase USP15 compared with other deubiquitinases. Should a dimeric UbV library be available for screening, we anticipate that this approach may be useful for a number of applications, particularly for elucidating and manipulating the activity of hetero- and homodimeric RING E3s.

## STAR★Methods

### Key Resources Table

**Table:** 

REAGENT or RESOURCE	SOURCE	IDENTIFIER
**Bacterial and Virus Strains**		

*Escherichia coli* BL21(λDE3) GOLD	Laboratory stock	N/A

**Chemicals, Peptides, and Recombinant Proteins**		

2-mercaptoethanol 99% 14.3 M (BME)	Sigma-Aldrich	Cat#M3148
Dithiothreitol (DTT)	Formedium	Cat#DTT025
Glutathione, reduced, free acid	Fisher Scientific UK	Cat#11483074
Imidazole BioUltra	Sigma-Aldrich	Cat#56749
Isopropyl-β-D-1-thiogalactoside (IPTG)	Formedium	Cat#IPTG025
Phenylmethanesulfonyl fluoride (PMSF)	Sigma-Aldrich	Cat#P7626
Ub74 and variants	(Gabrielsen et al., 2017) This paper	N/A
SUMO2-91 and variants	This paper	N/A
NEDD8-74 and variants	This paper	N/A

**Critical Commercial Assays**		

Morpheus screen	Molecular Dimensions	Cat#MD1-46

**Deposited Data**		

Ub74 G10V	This paper	PDB: 6QK9
UbV.XR from BIRC4 (XIAP) RING in complex with dimeric ubiquitin variant	(Gabrielsen et al., 2017)	PDB: 5O6T
UbV.XR / UbV.B4R a dimeric ubiquitin variant binding to BIRC4 (XIAP) RING	(Gabrielsen et al., 2017)	PDB: 5O6S
Ub	(Vijay-Kumar et al., 1987)	PDB: 1UBQ
K48-diUb from MINDY-1 tMIU in complex with K48-diUb	(Kristariyanto et al., 2017)	PDB: 5MN9
Chain B of Met1-diUb from OTULIN OTU domain (C129A) in complex with Met1-di ubiquitin	(Keusekotten et al., 2013)	PDB: 3ZNZ
SAXS data	This paper	https://doi.org/10.17632/wbbjg5kwr8.1

**Recombinant DNA**		

pGEX4T1 HG TEV Ub74	(Gabrielsen et al., 2017)	N/A
pGEX4T1 HG TEV Ub74 G10V	This paper	N/A
pGEX4T1 HG TEV Ub74 G10A	This paper	N/A
pGEX4T1 HG TEV Ub74 G10L	This paper	N/A
pGEX4T1 HG TEV Ub74 G10M	This paper	N/A
pGEX4T1 HG TEV Ub74 G10F	This paper	N/A
pGEX4T1 HG TEV Ub74 G10W	This paper	N/A
pGEX4T1 HG TEV Ub74 T7G G10V	This paper	N/A
pGEX4T1 HG TEV Ub74 K6G G10V	This paper	N/A
pGEX4T1 HG TEV Ub74 V5G G10V	This paper	N/A
pGEX4T1 HG TEV Ub74 F4G G10V	This paper	N/A
pGEX4T1 HG TEV Ub74 G10V K11G	This paper	N/A
pGEX4T1 HG TEV Ub74 G10V T12G	This paper	N/A
pGEX4T1 HG TEV Ub74 G10V I13G	This paper	N/A
pGEX4T1 HG TEV Ub74 G10V T14G	This paper	N/A
pGEX4T1 HG TEV Ub74 G10V L15G	This paper	N/A
pGEX4T1 HG TEV Ub74 G10V E16G	This paper	N/A
pGEX4T1 HG TEV NEDD8 74	This paper	N/A
pGEX4T1 HG TEV NEDD8 74 G10V	This paper	N/A
pGEX4T1 HG TEV SUMO2 91	This paper	N/A
pGEX4T1 HG TEV SUMO2 91 G27V	This paper	N/A
pGEX4T1 HG TEV SUMO2 91 G24A G27V	This paper	N/A

**Software and Algorithms**		

GraphPad Prism version 7	GraphPad Software	http://www.graphpad.com; RRID: SCR_002798
xia2 pipeline	(Winter, 2010)	http://xia2.github.io; RRID: SCR_015746
XDS	(Kabsch, 2010)	http://xds.mpimf-heidelberg.mpg.de/; RRID: SCR_015652
AIMLESS	(Evans and Murshudov, 2013)	http://www.ccp4.ac.uk; RRID: SCR_015747
MoRDA	(Vagin and Lebedev, 2015)	https://ccp4serv7.rc-harwell.ac.uk/ccp4online/; RRID: SCR_007255
PHENIX	(Adams et al., 2010)	https://www.phenix-online.org/; RRID: SCR_014224
COOT	(Emsley et al., 2010)	https://www2.mrc-lmb.cam.ac.uk/personal/pemsley/coot/; RRID: SCR_014222
MOLPROBITY	(Chen et al., 2010)	http://molprobity.biochem.duke.edu/; RRID: SCR_014226
ALINE	(Bond and Schüttelkopf, 2009)	http://bondxray.org/software/aline.html; RRID: SCR_016886
PyMOL	The PyMOL Molecular Graphics System, v. 1.8.4.0, Schrodinger, LLC	http://www.pymol.org; RRID: SCR_000305
SCÅTTER	SCÅTTER Version 3.1r	http://www.bioisis.net; RRID: SCR_017271
FoXS	(Schneidman-Duhovny et al., 2013)	https://modbase.compbio.ucsf.edu/foxs/; RRID: SCR_017269
DAMAVER	(Volkov and Svergun, 2003)	https://www.embl-hamburg.de/biosaxs/damaver.html; RRID: SCR_015648
SASRES	(Tuukkanen et al., 2016)	https://www.embl-hamburg.de/biosaxs/software.html; RRID: SCR_015648
DAMFILT	(Volkov and Svergun, 2003)	https://www.embl-hamburg.de/biosaxs/damaver.html; RRID: SCR_015648
GASBOR	(Svergun et al., 2001)	https://www.embl-hamburg.de/biosaxs/gasbor.html; RRID: SCR_015648

**Other**		

Glutathione agarose resin	Web Scientific	Cat#ABT 4B-GLU-100
Ni^2+^ agarose resin	Web Scientifc	Cat#ABT 6BCL-QHNi-100
SD75 26/60 size-exclusion chromatography column	GE Healthcare	Cat#28-9898-34
SD75 10/300 analytical size-exclusion chromatography column	GE Healthcare	Cat#17-5175-01
Protein Thermal Shift Kit	Applied Biosystems, Thermo Fisher Scientific	Cat#4461146
7500 FAST RT-PCR machine	Applied Biosystems, Thermo Fisher Scientific	N/A

### Lead Contact and Materials Availability

Further information and requests for resources and reagents should be directed to and will be fulfilled by the Lead Contact, Danny T. Huang (d.huang@beatson.gla.ac.uk).

### Experimental Model and Subject Details

#### Recombinant Proteins

Recombinant proteins were overexpressed in *Escherichia coli* BL21(λDE3) GOLD cells. Cultures were grown at 37°C in Luria Bertani media until an OD_600_ of 0.6–0.8 was reached, followed by induction with 0.2 mM isopropyl β-D-1-thiogalactopyranoside and incubation at 18–20°C overnight.

### Method Details

#### Generation of New Constructs

β1-β2 loop variants of Ub and Ubls were generated using standard PCR-ligation techniques and verified by automated sequencing. All UbVs and Ubls were cloned into pEX4T1 (GE Healthcare) modified with a TEV cleavage site and an N-terminal His-tag (pGEX4T1 HG TEV) and include DNA encoding the sequence GGS at the N-terminus prior to Met1 but lacking the C-terminal diglycine.

#### Protein Purification

After expression, cell cultures were harvested by low-speed centrifugation, resuspended in 50mM Tris-HCl pH 7.6, 200 mM NaCl, 2.5 mM phenylmethanesulfonyl fluoride (PMSF) and 15 mg lysozyme per liter culture, before being flash frozen in liquid nitrogen. The pellets were thawed and sonicated. Lysates were cleared by centrifugation at 50,000 *g* and then loaded onto glutathione agarose resin, followed by incubation for 1-2 hours. The proteins were washed in 50 mM Tris-HCl pH 7.6, 200 mM NaCl, 5 mM β-mercaptoethanol (BME), and eluted in 25 mM Tris-HCl pH 7,6, 150 mM NaCl, 5 mM BME, 10 mM glutathione. TEV protease was used to cleave the affinity tags, which were removed by further Ni^2+^-affinity chromatography. The proteins were concentrated to 5 ml, and applied onto a 26/60 SD75 gel filtration column, pre-equilibrated in 25 mM Tris-HCl pH 7.6, 150 mM NaCl, 1 mM DTT. All purification steps were carried out at 4°C.

#### Analytical Gel Filtration

Ub74 and variants were applied onto a Superdex 75 10/300 column pre-equilibrated in 25 mM Tris-HCl, pH 7.6, 150 mM NaCl, 1 mM DTT at constant flow-rate at 4°C.

#### Differential Scanning Fluorimetry (DSF) Assays

Proteins were mixed with buffer and ROX dye using the Protein Thermal Shift Dye Kit and thermal scanning (25 to 95°C at 1°C per minute) was performed using an Applied Biosystems 7500 FAST RT-PCR machine. Assay reactions were set up in triplicate in a 96-well PCR plate. Curve fitting and melting temperature calculations were performed using the basic Boltzmann sigmoid function in GraphPad Prism (version 7).

#### Small-Angle X-Ray Scattering (SAXS) Data Collection and Analyses

Protein was brought to Diamond Light Source station B21 for bioSAXS. Data were collected on a range of protein concentrations for both Ub74 G10V (from 50 mg/ml to 3.1 mg/ml) and Ub74 (from 25 mg/ml to 3.1 mg/ml). The protein samples were kept chilled until injected into the x-ray beam. Data were processed and the distance distribution function and maximum distance (D_max_) were calculated using SCÅTTER version 3.1r (http://www.bioisis.net).

Theoretical scattering curves were calculated and fitted against available crystal structures using FoXS (Schneidman-Duhovny et al., 2013). Twenty a*b initio* models were calculated by GASBOR (Svergun et al., 2001) and average models of these were calculated using DAMAVER and DAMFILT (Volkov and Svergun, 2003). The resolution of the space-filled models was calculated using SASRES (Tuukkanen et al., 2016).

#### Crystallization

Ub74 G10V was concentrated to approximately 50 mg/ml, and mixed in a 1:1 ratio of reservoir. Long rods, appearing within 24 hours, were grown using sitting drop vapour diffusion in 96 well trays. Initial crystals hits occurred in Morpheus HT-96 screen, condition 1. Final crystals were grown using hanging drop vapour diffusion in 24 well trays, where protein was mixed with the reservoir at 2:1 ratio. Furthermore, Buffer System 1 from the original condition was substituted with 1M MES pH 6.5.

#### Structure Determination and Refinement

Crystals were frozen without additional cryo-protection and brought to Diamond Light Source station I04-1. Data were collected in 0.2° slices, and processed by the XIA2 pipeline, relying on XDS (Kabsch, 2010) and AIMLESS (Evans and Murshudov, 2013). The structure was determined by molecular replacement using the MoRDA server (Vagin and Lebedev, 2015), which utilized a library of structures of ubiquitin from the PDB, finally determining the structure using chain B from PDB ID 3ZNZ. Refinement was done in PHENIX (Adams et al., 2010), with inspection of the maps and manipulation of the model in COOT (Emsley et al., 2010). Waters were added in COOT. Polder density maps were calculated in PHENIX and used to confirm the model.

### Quantification and Statistical Analysis

Table 1 and Figure 2 contain quantitative parameters related to data and refinement statistics for X-ray crystallography and SAXS experiments. Melting temperatures related to Figure 1E were obtained from the inflection point of melting curves measured in triplicate and fitted with the basic Boltzmann sigmoid function in GraphPad Prism (version 7). Normalization of absorbance on gel filtration chromatograms (Figures 1, 3, and S1) was performed using the following equation:

$$\;A_{norm}\;=\frac{\left(A-A_{min}\right)}{\left(A_{max}-A_{min}\right)}$$

### Data and Code Availability

The accession number for atomic coordinates and structure factors for the X-ray crystal structures of Ub74 G10V is PDB: 6QK9. SAXS data are available in the Mendeley Data Repository (https://doi.org/10.17632/wbbjg5kwr8.1). Other data generated in this study are available from the Lead Contact upon request.

## Supplemental Information

Document S1. Figure S1

Document S2. Article plus Supplemental Information
