# Supplementary material for: Identification and Characterization of Mutations in Ubiquitin Required for Non-covalent Dimer Formation
Source: Structure. 2019 Sep 3;27(9):1452–1459.e4. doi: 10.1016/j.str.2019.06.008 (PMC6720194; doi:10.1016/j.str.2019.06.008)
Supplement: Document S1. Figure S1 [file mmc1.pdf]

Structure, Volume *27*

Supplemental Information

Identification and Characterization of Mutations in Ubiquitin Required for Non-covalent Dimer Formation

Mads Gabrielsen, Lori Buetow, Dominika Kowalczyk, Wei Zhang, Sachdev S. Sidhu, and Danny T. Huang

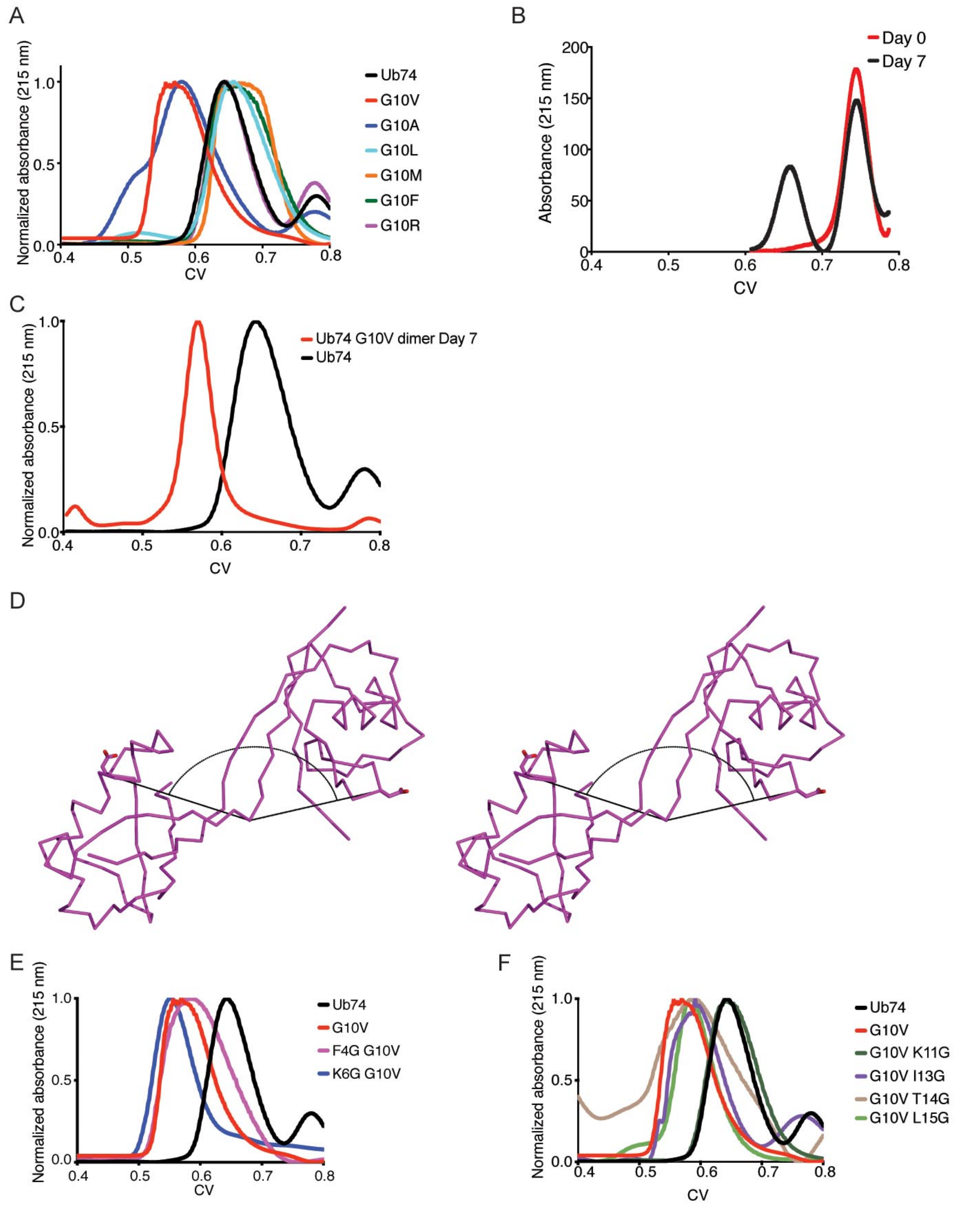

Supplementary Figure 1

(A) Normalized analytical size-exclusion chromatograms of Ub74 and Gly10-substituted variants. The profiles of Ub74 G10A and G10V have peaks that elute at volumes consistent with a dimer, whereas all other variants elute at volumes consistent with monomers. Related to **Figure 1C**.

(B) Analytical size-exclusion chromatograms of monomeric Ub74 G10V fraction after purification (Day 0; red line), and after 7 days (black line) at 4 °C. The x-axis differs from the other figures, as this analysis was performed on a new FPLC system with a new column. Related to **Figure 1D**.

(C) Normalized analytical size-exclusion chromatograms of purified dimeric Ub74 G10V left at 4 °C for 7 days (red line) and Ub74 (black line) for comparison. Related to **Figure 1D**.

(D) Superposed Cα-ribbon trace of chain CD (magenta) dimer from the crystal structure of Ub74 G10V, in wall-eye stereo. Asp39 and Val10 from chain C and Asp39' from chain D are shown as sticks. The measured angle is indicated. Related to **Figure 3A**.

(E) Normalized analytical size-exclusion chromatography of Ub74 G10V and Ub74 compared to Ub74 G10V variants in which residues in β1 have been replaced with Gly. Related to **Figure 3C**.

(F) As in (E) but compared to variants in which residues in β2 have been replaced with Gly. Related to **Figure 3C**.
